# Effect of Estragole on Leukocyte Behavior and Phagocytic Activity of Macrophages

**DOI:** 10.1155/2014/784689

**Published:** 2014-07-23

**Authors:** Francielli Maria de Souza Silva-Comar, Luiz Alexandre Marques Wiirzler, Saulo Euclides Silva-Filho, Raquel Kummer, Raissa Bocchi Pedroso, Ricardo Alexandre Spironello, Expedito Leite Silva, Ciomar Aparecida Bersani-Amado, Roberto Kenji Nakamura Cuman

**Affiliations:** ^1^Department of Pharmacology and Therapeutics, State University of Maringá, Avenida Colombo 5790, 870020-900 Maringá, PR, Brazil; ^2^Department of Clinical Analyses, State University of Maringá, 870020-900 Maringá, PR, Brazil; ^3^Department of Chemistry, State University of Maringá, 870020-900 Maringá, PR, Brazil

## Abstract

Estragole, a chemical constituent of the essential oils of many aromatic plants, is used as flavoring in beverage and food industries. *In vivo* and *in vitro* experimental assays have shown that EST has sedative, anticonvulsant, antioxidant, antimicrobial, and anesthetic activity. In this work, we evaluate the effect of EST on leukocyte behavior and phagocytic activity of macrophages. In the peritonitis model, EST (500 and 750 mg/kg) decreased the infiltration of peritoneal exudate leukocytes. *In vitro* chemotaxis assay showed that EST (3, 10, 30, and 60 *μ*g/mL) inhibited neutrophil migration toward fMLP. In the *in vivo* microcirculation assay, EST at doses of 250, 500, and 750 mg/kg significantly reduced the number of rolling and adherent leukocytes and at doses of 250 and 500 mg/kg decreased number of leukocyte migrated to perivascular tissue. The results showed that EST (3, 10, and 30 *μ*g/mL) was able to stimulate the macrophages phagocytosis but only at concentration of 10 *μ*g/mL promoted an increase in nitric oxide (NO) production. In conclusion, this study showed that EST had potential anti-inflammatory effects, likely by inhibiting leukocyte migration and by stimulating macrophages phagocytosis.

## 1. Introduction

Estragole (EST) is a monoterpene that is largely used in the food and beverage industry as a flavoring and also in the perfumes, soap, and detergents. It is an important chemical constituent of the essential oils of many aromatic plants, such as* Croton zehntneri *Pax et Hoffm.,* Artemisia dracunculus *(Asteraceae),* Ocimum basilicum* (Lamiaceae),* Pimpinella anisum* (Apiaceae),* Illicium anisatum* (Illiciaceae), and* Foeniculum vulgare *(Apiaceae) [1, 2].

*In vivo* and* in vitro* experimental assays have shown that EST has sedative, anticonvulsant, antimicrobial, and antioxidant activities, affects central nervous system, and acts as nervous excitability blocker in a concentration-dependent manner and in neuronal excitability by direct inhibition of Na^+^ channels [3–6]. Anethole, a position isomer of EST, showed anti-inflammatory properties as demonstrated in carrageenan-induced pleurisy in rats, inhibited TNF-induced inflammation, and reduced the inflammatory pain in models of carrageenan-induced paw edema and mechanical hypernociception [7–9]. Although several studies have demonstrated the effects of EST, there are few reports in the literature of the anti-inflammatory activity of this compound. Only its antiedematogenic effect on acute paw edema in mice was demonstrated [10].

The acute inflammation is a process characterized by a vascular response and initial recruitment of polymorphonuclear cells, typically neutrophils, followed by monocytes, which differentiate into macrophages. Neutrophils, via expression of adhesion molecules, adhere to the endothelium and migrate to reach the site of injury [11, 12]. The inflammatory reaction is necessary for tissue recovery and provides the correct cytokine signals and cell machinery to clear up the site for tissue regeneration. However, uncontrolled inflammation accompanied by the excessive migration of leukocytes has unfavorable effects on the course of tissue healing due to proteolytic enzymes release and reactive oxygen species production [13].

The present study was performed to evaluate the anti-inflammatory activity of EST in different models of acute inflammation. Specifically it has measured the carrageenan-induced peritonitis in mice,* in vivo* leukocyte migration in rats,* in vitro* chemotaxis, cytotoxicity, and phagocytic activity assay.

## 2. Materials and Methods

### 2.1. Chemicals

Estragole, indomethacin, zymosan, LPS (lipopolysaccharides from* Salmonella enterica* serotype* typhimurium*), fMLP (formyl-methionyl-leucyl-phenylalanine), and carrageenan were purchased from Aldrich Chemical Co.

### 2.2. Animals

Male Wistar rats (180–220 g) and male mice BALB-c (20–25 g) were maintained under a controlled temperature of 22°C on a 12 h light/dark cicle and receiving food and water* ad libitum*. The experimental protocols were approved by the Ethical Committee in Animal Experimentation of the State University of Maringá (CEAE/UEM 126/2010).

### 2.3. Peritonitis Model

BALB-c mice, in groups of five animals to each dose, were orally treated with EST (250, 500, or 750 mg/kg) 30 min before the intraperitoneal injection carrageenan solution (500 *μ*g/mice). The animals were euthanized 4 h later and the peritoneal cavity was washed with 2 mL of phosphate-buffered saline (PBS) that contained ethylenediaminetetraacetic acid (EDTA). The leukocyte count was determined in the fluid recovered from the peritoneal cavity. The results obtained in the differential count were expressed as the number of neutrophils per cavity.

### 2.4. *In Vivo* Leukocyte Migration

Rolling and adhesion of leukocytes to the endothelium were evaluated in the rat internal spermatic fascia 2 h after carrageenan injection (100 *μ*g) in the wall of the scrotal chamber. EST (250, 500, or 750 mg/kg), indomethacin (5 mg/kg), or saline (0.9%) was administered orally 30 min before carrageenan injection to different groups of rats (*n* = 5–7 animals/group). Animals anesthetized with chloral hydrate (600 mg/kg, s.c.) were maintained on a special board thermostatically controlled at 37°C with a transparent platform for transillumination of the tissue on which the spermatic fascia was exposed and fixed for analysis by microscopy* in situ*. The preparation was kept moist and warm with Ringer-Locke's solution (pH 7.2–7.4) containing 1% gelatin. The vessels selected for the study were postcapillary venules with a diameter of 15–25 *μ*m. The number of rolling and adherent leukocytes was determined at 10 min intervals. The leukocytes were considered to adhere to the venular endothelium if they remained stationary for more than 30 s. In another series of experiments, the number of leukocytes that migrated to an area of 2,500 *μ*m^2^ of connective tissue adjacent to postcapillary venules 4 h after carrageenan injection was determined. The number of cells was determined by an image recorded using four different fields for each animal, and the average value was calculated.

### 2.5. Chemotaxis Assay

To evaluate the effects of EST on chemotaxis, neutrophils were obtained 4 hours after injection of solution of zymosan in saline (1 mg/cavity) into the peritoneal cavity of BALB-c mice. The cell number was adjusted to 1 × 10^6^ cells/mL in RPMI medium that contained 0.1% bovine serum albumin (BSA). The chemotaxis assay was performed using a 48-well microchemotaxis plate (Neuro Probe), in which the chambers were separated by a polyvinylpyrrolidone-free polycarbonate membrane (5 *μ*m pore size). The chemoattractant *N*-formyl methionyl leucyl phenylalanine (fMLP; 10^−6^ M) and a negative control (RPMI 1640) were placed in the lower chamber. A neutrophil suspension (1 × 10^6^ cells/mL) pretreated with EST (3, 10, 30, or 60 *μ*g/mL) for 30 min was then placed in the upper chamber. The cells were allowed to migrate into the membrane for 1 h at 37°C in 5% CO_2_. After incubation period, the membrane was washed and stained using Instant Prov (Newprove). The membrane area of each well was scored using light microscopy to count the intact cells present in five random fields. The results were expressed as the mean number of neutrophils per field and representative of three separate experiments.

### 2.6. Phagocytic Assay

Peritoneal macrophages from male BALB-c mice (20–25 g) were collected with precooled phosphate buffer saline (PBS), harvested in RPMI 1640 medium pH 7.6 supplemented with 10% FBS (5 × 10^5^ cells/mL), and plated on 13 mm coverslips in 24-well culture plates for 1 h at 37°C in a 5% CO_2_ atmosphere. Nonadherent cells were then removed and adhered to macrophages cultured with EST (3, 10, 30, or 60 *μ*g/mL) in RPMI 1640 medium supplemented with 10% FBS for 24 h at 37°C in a 5% CO_2_. After this, treated macrophages were challenged with multiples of 10 chicken red blood cells (CRBC) for each macrophage and incubated for 1 h at 37°C in a 5% CO_2_ atmosphere. The monolayers were fixed with xylol and stained with panótico. The number of phagocytized CRBC was determined by counting at least 100 macrophages in duplicate cultures, and results were expressed as the phagocytosis index (PI), the percentage of infected macrophages by the mean number of phagocytized CRBC per cell. Cell viability was ≥90% in all experiments. LPS (lipopolysaccharide) at 20 *μ*g/mL was used as positive control.

### 2.7. Measurement of Nitric Oxide (NO) in Macrophages

The level of NO production was monitored by measuring the nitrite level in the culture medium by Griess reaction. Cell free supernatants (50 *μ*L) were incubated with equal volumes of Griess reagent mixtures (1% sulfanilamide in 5% phosphoric acid and 0,1% N-1-naphthylethylenediamine dihydrochloride in water) at room temperature for 10 min. The absorbance was measured in a microplate reader at 550 nm. NO concentrations were calculated from a sodium nitrite standard curve. Data were presented as *μ*M concentration of NO^2−^.

### 2.8. Dimethylthiazol Diphenyltetrazolium Bromide (MTT) Assay

The tetrazolium reduction assay was done as described by Denizot and Lang [14]. Peritoneal macrophages were isolated as described above. The macrophages were incubated for 24 h with EST (3 *μ*g/mL, 10 *μ*g/mL, 30 *μ*g/mL, or 60 *μ*g/mL). The supernatant was then removed and macrophages were incubated for 3 h with medium RMPI containing 5 mg of MTT/mL. A hundred *μ*L of DMSO was added in each well. The cells were incubated for 10 min at 25°C. The resulting absorbance was measured at 540 nm.

### 2.9. Statistical Analysis

Results are expressed as mean ± standard error of the mean (SEM). Data were subjected to analysis of variance (ANOVA) followed by Tukey's post hoc test. Values of *P* < 0.05 were considered statistically significant.

## 3. Results and Discussion

The acute inflammatory process is characterized by vasodilation, exudate formation, and leukocyte migration. The migration and accumulation of neutrophils at the inflammation site are crucial for host defense and these processes are mediated by a signaling cascade that involves adhesion molecules, several cytokines, and production of nitric oxide [15, 16]. However, a persistent leukocyte migration may damage the surrounding tissue through the release of proteolytic enzymes and reactive metabolites of oxygen and nitrogen [17]. Thus, drugs that modulate leukocyte recruitment may provide an interesting therapeutic option for acute and chronic inflammation.

**Figure 1:**
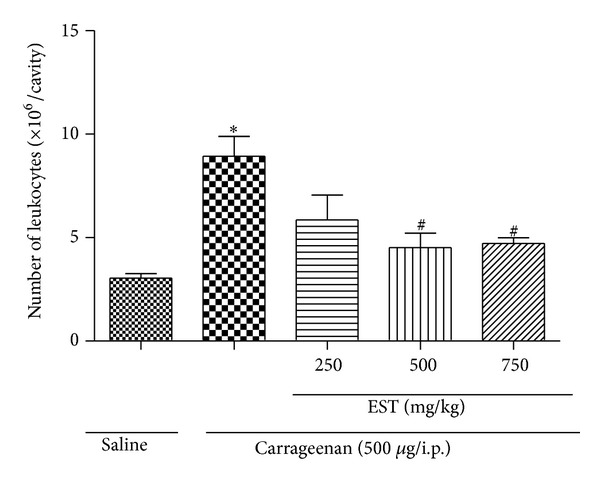
Effect of EST treatment on leukocyte number. Effect of treatments on leukocyte number 4 hours after carrageenan injection (500 *μ*g/mice i.p.) in BALB-c mice. Values are mean ± SEM (*n* = 5). *P < 0.05 versus saline (vehicle). ^#^ *P* < 0.05 compared versus carrageenan group (one-way ANOVA, Tukey's test).

The inflammatory activity of EST was evaluated in a murine carrageenan-induced peritonitis model, used to screen anti-inflammatory drugs, and the results obtained are shown in Figure 1. Carrageenan is a phlogistic agent widely used for the induction of an inflammatory response involving the participation of cytokines (TNF-*α* and IL-1*β*), arachidonic acid-derived mediators, and nitric oxide [18–20]. After 4 h of peritonitis induction, an inflammatory response was observed characterized by an increase in the number of leukocyte counts in the peritoneal cavity (8.90 ± 1.28 × 10^6^ cells/cavity) when compared with the control group (3.05 ± 0.20 × 10^6^ cells/cavity). The pretreatment of animals with EST at doses of 500 and 750 mg/kg significantly inhibited the leukocyte migration (51% and 52%, resp.), providing evidence that this compound presents activity on inflammation. There are some reports describing that monoterpene compounds, such as borneol, promote anti-inflammatory activity by reducing the leukocyte migration induced by carrageenan in peritonitis model [21]. Our data suggest that EST could have a similar effect on anethole, which was able to decrease leukocytes recruitment in carrageenan-induced pleurisy model, probably attributed to the inhibitory effect on production and/or release of nitric oxide and active metabolites of arachidonic acid [7].

**Figure 2:**
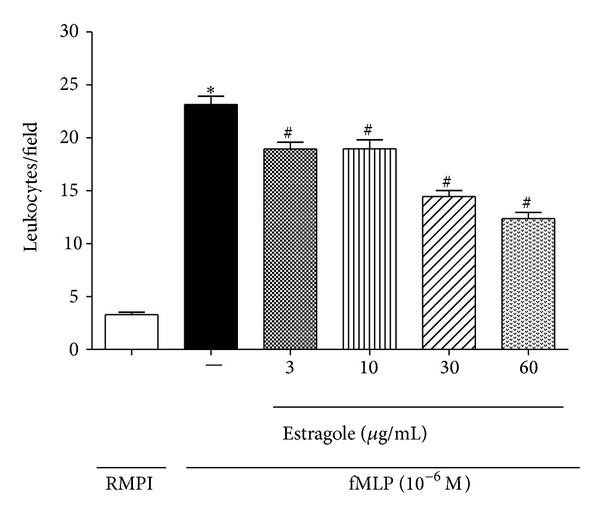
EST inhibited* in vitro* leukocyte chemotaxis. Leukocytes were obtained from zymosan-induced peritonitis (1 mg/cavity) in mice and stimulated with fMLP (10^−6 ^M) after 30 min of treatment with EST at doses 3, 10, 30, and 60 *μ*g/mL. Values are mean ± SEM (*n* = 5) and are representative of three independent experiments. *P < 0.05 versus medium (RPMI 1640); ^#^ *P* < 0.05 versus group of neutrophils stimulated with fMLP (one-way ANOVA, Tukey's test).

To verify the direct effect of EST on* in vitro* leukocyte chemotaxis, the study was performed using 10^−6^ M fMLP (formyl-methionyl-leucyl-phenylalanine) as chemotactic agent. fMLP is a chemotactic agent associated with the production/release of cytokines, mainly IL-1*β*, IL-8, and TNF-*α* [22]. The results obtained are shown in Figure 2. fMLP induced a significant leukocyte migration when compared with the control group (RPMI 1640). After leukocytes incubation with EST (3, 10, 30, and 60 *μ*g/mL) a significant reduction (18, 10%; 18, 02%; 37, 55%; 46, 54%, resp.; *P* < 0.05) in a dose-dependent manner of leukocyte migration in response to fMLP stimulation was observed. Since EST treatment did not affect the leukocytes viability evaluated by cytotoxicity assay at all concentrations tested (data not shown), our data suggest that the direct effect of EST on inhibition of leukocyte migration does not seem to be related to toxic effects (e.g., cell death). Recent study performed in our laboratory showed that both anethole and eugenol (structural analog of EST) reduced leukocyte migration induced by fMLP, probably by inhibitory effects of these compounds on synthesis/release cytokines [23].

**Figure 3:**
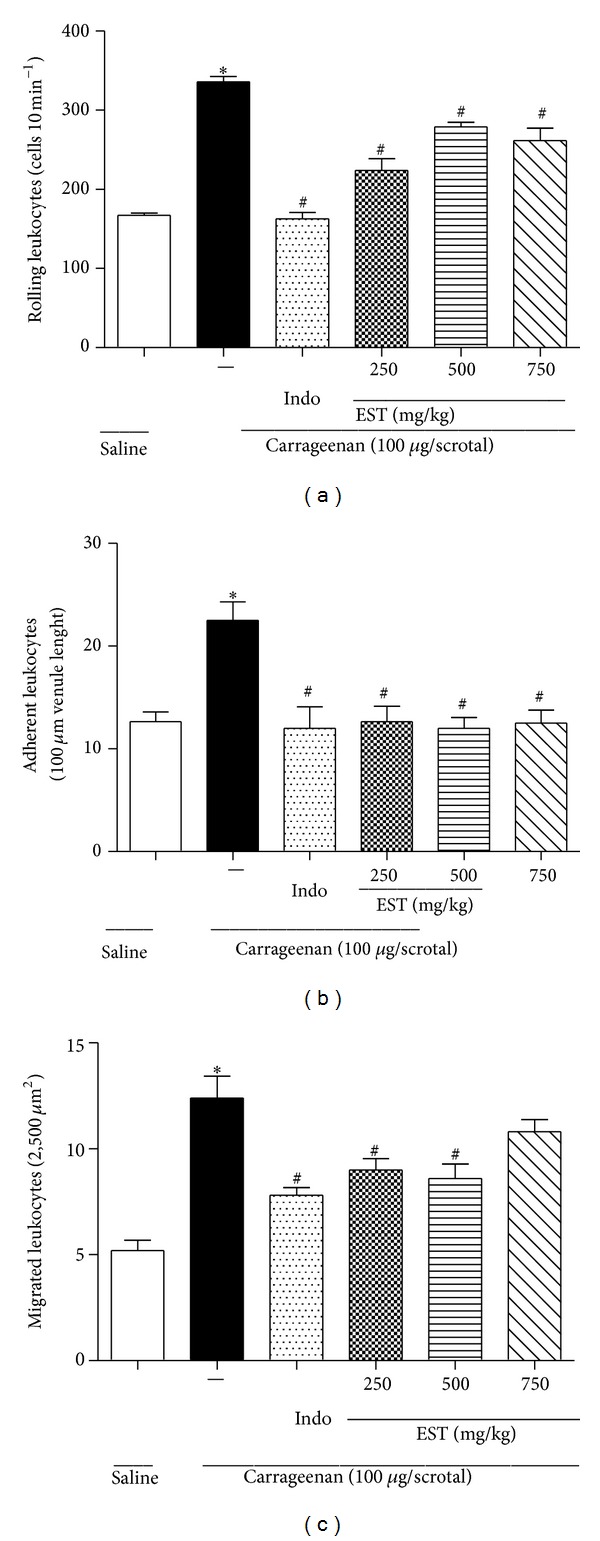
EST inhibited* in vivo* leukocyte migration in rats. The animals were orally treated with EST (250, 500, or 750 mg/kg) and indomethacin (5.0 mg/kg) 30 minutes before the injection of carrageenan (100 *μ*g/scrotum). (a) Number of leukocytes rolling, (b) number of leukocytes adherent during 10-minute periods after 2 h, and (c) leukocytes migrate after 4 h of inflammatory stimulus. Values represent mean ± SEM (*n* = 5–7 animals/group) and are representative of three independent experiments. *P < 0.05 versus vehicle; ^#^ *P* < 0.05 versus carrageenan-injected group (one-way ANOVA, Tukey's test).

Mechanisms by which the EST decreases the leukocyte migration to the inflammatory site were investigated using intravital microscopy system. EST was administered and the* in vivo* leukocyte-endothelial interactions (rolling, adhesion, and migration) were evaluated in the internal spermatic fascia postcapillary venules. During an inflammatory response, the leukocyte migration from the bloodstream to the site of injury occurs mainly in postcapillary venules by distinct events, such as rolling, adhesion, and transmigration. These processes are mediated by a sequence of interactions between the surface of leukocytes and endothelial cells, which require the presence of a variety of adhesion molecules, chemokines, and cytokines (TNF-*α* and IL-1*β*) [24–27]. Two hours after carrageenan injection (100 *μ*g/cavity) into the scrotum, a marked increase in the number of rolling and adherent leukocytes in the vascular endothelium was observed (Figures 3(a) and 3(b)). EST at all the doses tested (250, 500, or 750 mg/kg) caused a significant reduction of leukocytes rolling and this effect was more intense at the lower dose (33, 20%; 16, 92%; 22, 08%, resp.). We also observed that the number of adherent leukocytes was reduced by EST at all doses tested. In addition, the leukocytes migration into the perivascular tissue was significantly diminished by EST in doses of 250 and 500 mg/kg (Figure 3(c)). A similar effect was also verified after treatment with reference drug, indomethacin (5 mg/kg).

Some studies have suggested that herbal compounds of the terpene group possess anti-inflammatory activity by inhibiting the production of TNF-*α* and IL-2 [8, 12, 28, 29]. Recently it was demonstrated by our research group that anethole decreased the acute inflammatory process by inhibiting some mediators such as NO, PGE_2_, TNF, IL-1 *β*, and IL-17 and it was also able to inhibit leukocyte migration* in vitro* and* in vivo* [7, 9, 23]. Indeed, EST presented inhibitory activity on paw edema induced by TNF-*α* in mice [10]. Cytokines such as TNF and IL-1*β* promote leukocyte recruitment by inducing the integrins expression, the selectins expression on endothelial cells, and upregulation of ICAM expression [30–32]. Considering the structural similarity between anethole and EST, and the effects already described to anethole on inflammatory response, it is possible that EST could be acting in the adhesion molecules production by inhibiting TNF and IL-1*β* production, with consequent reducing in the migration of leukocytes. However, it is necessary to perform additional experiments to evaluate the action of EST on the adhesion molecules.

**Figure 4:**
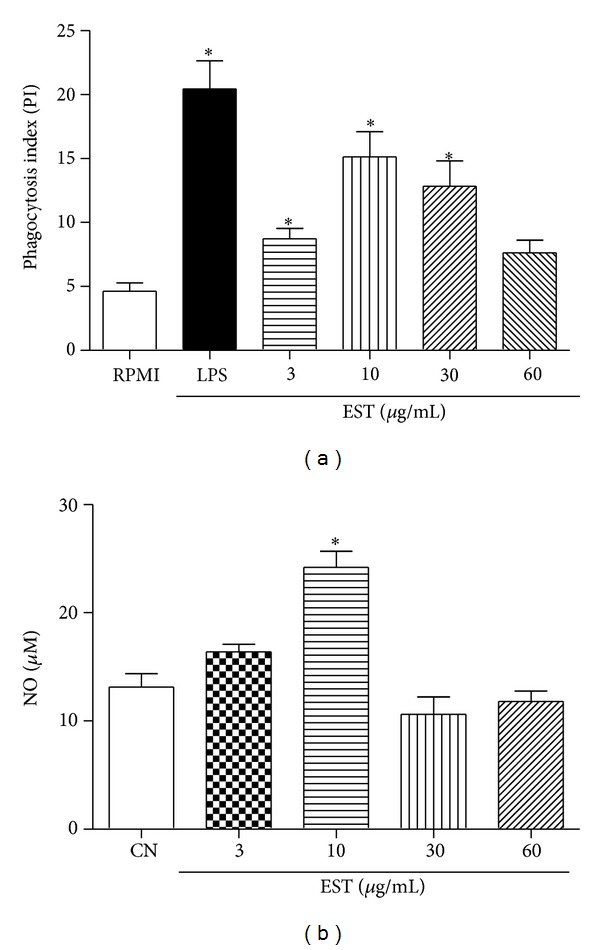
Effect of different concentrations of the EST on phagocytosis and NO production. (a) Phagocytosis index in macrophages. The peritoneal macrophages were incubated with EST (3, 10, 30, and 60 *μ*g/mL) for 24 hours at 37°C with 5% CO_2_. LPS 20 *μ*g/mL was used as positive control. (b) NO production in peritoneal macrophages. Values represent mean ± SEM (*n* = 5) and are representative of three independent experiments. *P < 0.05 versus negative control (one-way ANOVA, Tukey's test).

Inflammation resolution begins in the first hours after the initiation of the inflammatory response and it is primarily mediated by resident macrophages recruited from the tissues. Macrophages represent the first line of defense against pathogens and constitute the largest group of phagocytic leukocytes, which have a crucial role in the immune response of inflammatory and infectious diseases and their actions involve phagocytic activities and secretion of proinflammatory mediators. When macrophages are stimulated,* in vivo* or* in vitro*, they respond with increased production of NO and hydrogen peroxide (H_2_O_2_) that are highly toxic to microorganisms [33–35]. Since phagocytic activity is one of the most important functions of macrophages, the effect of EST on the phagocytic activity of macrophages was evaluated at concentrations of 3, 10, 30, and 60 *μ*g/mL. Our results demonstrated that EST stimulated significantly the macrophages phagocytic ability in concentrations of 3, 10, and 30 *μ*g/mL, when compared with negative control group (Figure 4(a)). A similar effect was observed when LPS was used as positive control. LPS, a component of the membrane of Gram-negative bacteria, activates macrophages by binding the Toll-like receptors 4 (CD14/TLR4) complex, which promotes phagocytosis [36]. Thus, EST can also have stimulated the phagocytosis via binding with Toll-like receptors 4, but additional experiments are still necessary to confirm the mechanism.

Cytotoxicity assays in macrophages were done using same concentrations as described in the experiments above. EST at concentrations of 3, 10, 30, and 60 *μ*g/mL showed cell viability of 86%, 87%, 87%, and 71%, respectively. Only at a concentration of 60 *μ*g/mL that EST did not stimulate the phagocytosis, probably due to the low cell viability (less than 75%).

NO is involved in several functions in the human body, such as blood pressure control, neurotransmission, and host defense [37]. Indeed, in the inflammation, NO participates in infection control, regulation of leukocyte rolling, migration, cytokine production, proliferation, and apoptosis [38]. It is well known that NO produced by macrophages is a cytotoxic molecule acting by a killing mechanism against invading microbes [35]. In the present work, different concentrations of EST (3 *μ*g/mL, 10 *μ*g/mL, 30 *μ*g/mL, and 60 *μ*g/mL) on the production of NO were evaluated in the culture supernatant of peritoneal mice macrophages. As shown in Figure 4(b), the addition of EST significantly stimulated production of NO only at the concentration of 10 *μ*g/mL, similar to other natural products, such as Actin A, concanavalin A, and phytohemagglutinin [36, 39].

## 4. Conclusion

In conclusion, EST showed anti-inflammatory activity as demonstrated by the inhibition in the leukocyte recruitment and stimulation of phagocytic activity in macrophages. Thus, this compound may be a promising herbal medication in inflammatory process with excessive leukocyte migration. This finding is relevant since EST is a constituent of many essential oils used in aromatherapy and could be considered as nutraceutical. Further studies are needed to elucidate this possibility and also the anti-inflammatory mechanism of this drug.
